# Call the experts: identifying stakeholders in the long-term care of youth with hypospadias

**DOI:** 10.3389/fped.2024.1445205

**Published:** 2024-10-30

**Authors:** Vinaya P. Bhatia, Kristin Ebert, Shannon Cannon, Walid A. Farhat, Heidi W. Brown, Jane Mahoney, Marisa E. Hilliard, Kristina L. Penniston

**Affiliations:** ^1^ Department of Urology, Division of Pediatric Urology, University of Wisconsin-Madison-UW Kids, Madison, WI, United States; ^2^ Department of Obstetrics and Gynecology, Division of Urogynecology, Kaiser Permanente, San Diego, CA, United States; ^3^ Department of Internal Medicine, Division of Geriatrics and Gerontology, University of Wisconsin-Madison, Madison, WI, United States; ^4^ Department of Pediatrics, Division of Psychology, Texas Children’s Hospital/Baylor College of Medicine, Houston, TX, United States

**Keywords:** hypospadias, stakeholders, engagement, holistic care, adolescent and youth

## Abstract

Long-term follow-up for individuals with hypospadias remains a critical area of need, yet evidence-based guidelines for such follow-up are lacking, and the role of involvement of relevant experts is not yet established. Using our hypospadias-specific health-related quality of life conceptual framework and a subsequent qualitative study of prepubertal males and parents of males with hypospadias, we identified potential priorities for long-term follow-up of youth with hypospadias. Using thematic codes from our patient and parent interviews, we searched PubMed for relevant articles and identified the specialties represented by all the authors of these articles. Our search strategy revealed consistent expertise across HRQOL themes and subthemes, including pediatric and adult urology, health psychology, psychiatry, endocrinology, genetics, and social work. Communication experts, as well as patients and families, were also represented in our literature search. Using these findings, we compiled a comprehensive list of potential stakeholders to inform the development of holistic care guidelines for individuals with hypospadias. By engaging these stakeholders, we aim to develop consensus-based, long-term follow-up guidelines and tools to address the evolving physical and psychosocial needs of people with hypospadias over a lifetime. The use of qualitatively derived thematic codes to search for relevant literature is an accessible approach to identifying relevant stakeholders. These findings underscore the importance of involving diverse, multidisciplinary expertise to ensure comprehensive, patient-centered care in complex genitourinary conditions.

## Introduction

Current longitudinal research suggests that long-term follow-up for youth and adults with hypospadias is required for close monitoring of physical and psychological complications after repair (1–3). However, no formal guidelines for long-term follow-up exist, and there is a paucity of literature on which specialties or experts should be involved in the long-term follow-up of this population. Research on improving the quality of care in chronic pediatric urologic diseases suggests that identifying experts who need to be involved in long-term care is a critical step to facilitate appropriate long-term follow-up (4).

**Figure 1:**
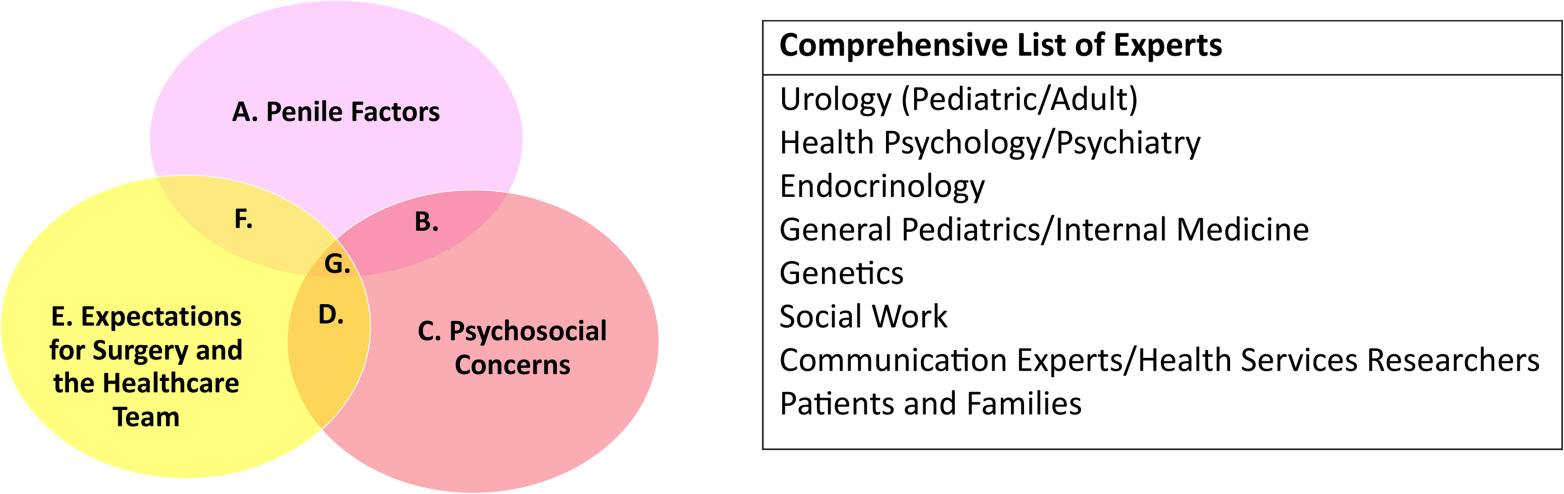
Themes and experts. Below, we include themes identified from the literature review and parent/youth interviews, as well as identified stakeholders for expert consensus.

Frameworks to identify appropriate experts in other urological conditions refer to the understanding of condition-specific health-related quality of life (HRQOL) (5) and use extensive patient input to identify and prioritize concerns (6). To apply these approaches to hypospadias, we previously published a novel conceptual framework for health-related quality of life (HRQOL) for youth with hypospadias based on a scoping literature review (7). This framework included themes of penile appearance, voiding function, social function, psychological and behavioral function, and sexual function. Our subsequent qualitative study of 8–12-year-old males and their parents supported the hypospadias-specific HRQOL Framework for prepubertal males (Figure 1, manuscript in review). Together, our previous work has synthesized youth and parent experiences after hypospadias repair, which are important to consider in identifying clinical priorities.

We hypothesized that we could identify a consistent and comprehensive list of potential experts and stakeholders by using our previously generated codes from qualitative interviews of people with hypospadias as our search terms. Our goal would then be to contact these potential expert stakeholders and attempt to establish consensus between healthcare providers and patients on priority concerns and an optimal timeframe for follow-up for different concerns.

## Methods

### Qualitative methods

We previously conducted an IRB-approved, semi-structured interview study of English-speaking 8–12-year-old males with a history of repaired hypospadias (8). Potential participants with medical or neurocognitive conditions that would prevent the ability to autonomously answer interview questions were excluded. We completed interviews for 20 families (18 individuals, 10 adults parents, 8 youth). Interviews were conducted over the telephone, audiorecorded, transcribed, and de-identified. We employed hybrid thematic analysis to generate a first-round codebook to describe patient experiences, parent–proxy experiences, and parent experiences. Interviews were continued until thematic saturation was achieved (i.e., no new concepts or codes were generated). The second round of value-based coding was then performed with two investigators. The final thematic analysis was completed using the second-round coding with expertise from the listed authors in qualitative research, pediatric urology, and psychology. All coding was completed in NVivo 12 software (QSR International, 2020). Highest-order themes and subthemes were confirmed by the study investigators (VPB, SC, ME, HWB, WAF).

### Search approach and terms

To identify expertise related to hypospadias-specific HRQOL, we used the domains of our published HRQOL framework (7) and the themes and subthemes of our qualitative research as search terms in PubMed. Specifically, the three overarching themes were penile factors, psychosocial concerns, and expectations of surgery and the healthcare team (Figure 1), each of which had subthemes that are listed in Table 1 (8). The duration of inclusion of our search was 15 years (December 2008–December 2023). PubMed was selected as the search engine of choice for article identification and selection due to the precision of search results, reliability of the search algorithms for future reproducibility of our methods, and higher utility of clinically oriented searches based on existing studies (9, 10). The word “hypospadias” was added to each search term to improve the specificity of our search. Articles were reviewed for relevancy to hypospadias in each search by the first author. Engagement of physicians, parents, or youth and adults with hypospadias was also collected for the included studies and authors (Table 1). Article titles and author affiliations and expertise were then disseminated to the author list for approval prior to the final analysis and compilation of the potential expert list.

**Table 1:** List of subthemes, references, engaged parties, and represented specialties.

Subthemes/search terms	Sample reference	Study type	Patient/parent or physician actively engaged in research?	Specialities represented across publications
Penile factors				
A. Penile function	Hadidi AT, et al. (2023)	Retrospective cohort study	No	Pediatric urology Pediatric endocrinology
B. Urinary spraying/control, penile appearance (discomfort with, embarrassment, penile size comparison)	Rourke K, et al. (2018) Gulseth E, et al. (2023) Schober JM, et al. (2009) Ryu DS, et al. (2018) Stancampino, et al. (2022)	Case series Valid PROM, qualitative Interview Non-validated survey study Prospective cohort study Scoping literature review	No 16-year-old patients with distal HS and health adolescents 22–57 year-old adult males with no genital surgery hx Infant males ages 6–24 months in Korea with cryptorchidism No	Pediatric urology Adult reconstructive urologist Child psychiatry/health psychology Pediatric endocrinology
Psychosocial concerns				
C. Social functions (stigma/disclosure, interpersonal interactions/teasing), future worries, public restrooms, growth/development (hypospadias)	Media LM, et al. (2022) Cheng JW, et al. (2022) Lee P, et al. (2012) Blankstein, U. (2022) Rolston AM, et al. (2015) Ortqvist L, et a. (2019) Bhatia VP, et al. (2021) Toufaily, et al. (2018)	Qualitative interview Observational study (social media) Scoping literature review Retrospective cohort study Prospective survey study Prospective survey study Scoping literature review Retrospective cohort study	18–35 year-old adults with DSD No No No Parents of children with DSD age 6–9 months (Study 1) or 8–17 years (Study 2) Adult males in Sweden (no age specified) with hypospadias No No	Pediatric urology Pediatric endocrinology Child psychiatry General pediatrics Health psychology/health psychiatry Genetics
D. Trauma from surgery	Duarsa, GK, et al (2019)	Case control, prospective survey study	Children with a history of repaired hypospadias (age not specified)	Pediatric urology Psychology/psychiatry
Expectations for surgery and the healthcare team				
E. Setting expectations, no knowledge of surgery, provider experience, education/advocacy	Phillips L, et al. (2023) Chan KH, et al. (2020) Chan KH, et al. (2020) Rourke, et al. (2018)	Qualitative interview study Qualitative interview study Focus group study of providers Case series	Adult males age 20–49 with previous hypospadias surgery Parents of youth <18 with history of hypospadias Physicians (urologists, pediatricians) No	Pediatric urology Social work Patient-provider communcation experts/health services researchers Pediatrics Endocrinology
F. Surgical outcomes, benefits of surgery	Keays, M et al. (2016)	Qualitative interview and survey study	Caregivers of children <8 years old children >8 years old	Pediatric urology, psychology
Central overlaps				
G. Uncertainty, complications, social disadvantage, worries (about fertility, normality, embarrassment, complications)	Chan KH, et al. (2020) van der Horst, HJR, et al. (2017) Chang, E, et al. (2022) Snodgrass P, et al. (2021) Gul M, et al. (2021) van der Horst, (2017)	Qualitative interview study Literature review Retrospective cohort study Survey study Narrative review Non-systematic review	Parents of youth <18 with history of hypospadias No No Parents of youth with hypospadias No No	Pediatric urology Patient-provider communcation experts/health services researchers Pediatrics Endocrinology Andrology

### Specialty determination and discrepancy resolution

For this exploratory study, a “potential expert” was defined as someone beyond the training phase of education (i.e., completed residency and/or fellowship) who had published at least one article as a first, middle, or senior author on the topic of hypospadias. We performed confirmation searches of the specialties of all listed authors in Google Scholar and ORCID (when available) to determine the correct specialty based on the author's most recent institution affiliation on publications within the last 5 years. At least two sources were required to confirm the author's expertise and affiliation at the time of publication, either two articles or one article and an ORCID (when available) that was linked to up-to-date references (within the past 5 years). For authors who were non-specialized trainees (i.e., undergraduate, medical students, residents, or fellows) at the time of publication, as confirmed by Google searches, the specialty of record was excluded. We generated an initial list of potential expert stakeholders for the holistic care of people with hypospadias from the list of represented specialties (Figure 1).

## Results

We identified at least one relevant article for each hypospadias-specific HRQOL theme and subtheme, as detailed in Table 1. The table also includes information on patient or parental participation and general authorship expertise for each relevant article.

### Penile factors

Penile functions refer to erections and voiding. Our search of “penile functions hypospadias” revealed articles authored mainly by pediatric urologists (11). The search of “urinary spraying hypospadias” elicited articles by pediatric and adult urologists (12). Searches regarding “discomfort with penile appearance hypospadias” (13, 14) included psychiatrists, endocrinologists, health psychologists, and pediatric urologists. A search regarding “hypospadias penile length” included authors from genetics and endocrinology (15, 16). Searches with the terms embarrassment and penile size comparison did not identify any articles. We did find that authors engaged the input of patients (pediatric and adult) in an attempt to understand the impacts of urinary spraying and control and penile appearance on quality of life.

### Psychosocial concerns

In the realm of psychosocial concerns, searches regarding “stigma hypospadias” yielded expertise from health psychologists (17), pediatric urologists (18), and pediatricians (19). A search of “future worries hypospadias” identified articles by health psychologists, psychiatrists, and pediatric urologists (20). A subsequent search of “interpersonal actions hypospadias” and “teasing hypospadias” referred to articles by pediatric urologists (21) and endocrinologists, psychologists, and geneticists (22). Searches related to “public restroom/public toilet hypospadias” referred to articles authored by psychologists and urologists (7), while searches surrounding “growth and development hypospadias” were authored by geneticists and pediatricians (23). The authors engaged adult patients, parents, and social media to perform their analysis

### Expectations of surgery and the healthcare team

When searching “knowledge of hypospadias surgery” and “provider experience hypospadias,” we identified two articles written by urologists, endocrinologists, and patient–provider communication experts (24, 25). When searching “setting expectations hypospadias surgery,” we found articles authored by pediatric urologists and social workers (26). A search for “education and advocacy hypospadias” did not yield any results. Researchers obtained these perspectives from adult and pediatric patients, parents, and physicians.

### Overlapping subthemes

When examining “trauma from hypospadias surgery” in the overlap of psychosocial functions and expectations of surgery, one article authored by urologists, psychologists, and psychiatrists was identified (27). A search focused on the overlap of expectations of surgery and penile factors was authored by psychologists and pediatric urologists (28).

Searches regarding “uncertainty hypospadias” led to an article previously identified from “knowledge of hypospadias surgery,” which was authored by pediatric urologists and patient-provider communication experts. A search regarding “social determinants of health hypospadias” was authored by pediatric urologists (29). Finally, searches regarding sources of “worry associated with hypospadias” drew articles with experts from pediatric urology (30, 31) and andrology (32).

## Discussion

This analysis illustrates a research-driven approach to the identification of appropriate experts for a complex and multifaceted disease. We used a conceptual framework for HRQOL and qualitative feedback from patient and parent stakeholders to identify clinical priorities and a wide range of clinical, research, and lived-experience expertise, which can be used to guide clinical care guidelines in this area. In addition, the ethnically, racially, and socioeconomically diverse demographics of our initial interview study should contribute to the broad applicability of our findings to English-speaking youth in the postoperative, prepubertal setting (8). Our search strategy successfully identified consistent expertise based on the search terms, as most of the article authors and experts were evident across multiple themes. This stepwise approach to identifying an array of potential stakeholders can prepare the field to develop comprehensive consensus-based holistic care guidelines, screening tools, and a referral network for youth and adults with hypospadias. Furthermore, this system for identifying stakeholders may be a useful approach for clinicians and researchers undertaking similar efforts in other complex, chronic genitourinary pediatric conditions, such as spina bifida, variations of sexual characteristics, or cloacal anomalies.

Engaging stakeholders in research requires identifying the appropriate domains of expertise (including not only clinical and research experts from various domains but also people with lived experience) in advance and adequately involving them in the development of research questions (4). Stakeholders may also help conduct studies and interpret results (4–6). Identifying all of the potential stakeholders is critical to ensure that the conducted research is of high quality, rigorous, and holistic (4), as well as relevant to the people impacted by the condition. Prominent examples of stakeholder identification in pediatric urology have included the use of a national patient advocacy network for care of spina bifida (6) and the creation of a partnership between physician and patient stakeholder core to iteratively revise and improve clinical trial design and surgical selection in pediatric kidney stone disease (33).

The current study extends this methodology to advance stakeholder engagement in research related to supporting HRQOL among people with hypospadias. Building on the results of this study that identified key stakeholders and important domains of expertise (Figure 1), our next goal will be to engage stakeholders in the design of screening tools and treatment strategies for hypospadias over the long term. Including our patient and parent experts will be critical to ensure that our study design is thorough and pragmatically addresses the lived experiences of youth and adults with hypospadias (5, 34).

Some stakeholders may bring expertise that is more relevant to specific stages of care for hypospadias than others. For example, an andrologist or adult reconstructive urologist may be particularly pertinent as the patient reaches sexual maturity (e.g., after puberty). Similarly, child psychology may need to be involved in care at the time point in development when body image, self-esteem, and interpersonal interactions are becoming a larger concern. Further qualitative study of patients in multiple different stages of development will be needed to ascertain these types of important details.

Limitations include the use of qualitative themes derived from interviews of patients at a specific time point in development (i.e., peri-pubertal), and the use of PubMed without other search engines to review available literature on our search terms. Additional research using interviews over multiple stages of development may identify additional themes and other experts not reflected in this study (e.g., adolescent medicine or sexual function/health). We acknowledge the importance of conducting such interviews in the near future to elucidate the needs of patients across a lifespan and ensure that appropriate experts are available to deliver optimal, long-term care. Our reliance on English language articles may exclude culturally relevant issues or expertise found in studies conducted in other regions of the world. Finally, this work is limited by the current state of the field—as research on medical, psychosocial, and healthcare delivery aspects of hypospadias care grows and innovates over time, this search should be repeated.

## Conclusions

Hypospadias-specific HRQOL is complex and may change over time as children become adolescents and then adults. Finding the experts to facilitate care and support patients over a lifetime requires a thoughtful approach to ensuring comprehensive multidisciplinary care. In this study, we identified potential stakeholders to be involved in the development of multidisciplinary care guidelines for long-term follow-up care of people with hypospadias, including urology (pediatric/adult), health psychology, psychiatry, endocrinology, general pediatrics, internal medicine, genetics, social work, communication experts/health services researchers, and patients and families. Our next goal will be to interview and survey these proposed specialists and patients across multiple stages of development (youth and adults) to obtain qualitative feedback on whether these proposed experts are needed for multidisciplinary care for youth and adults with hypospadias. Once we have obtained this additional feedback, we intend to use the revised list of experts to develop consensus-based guidelines for long-term follow-up after hypospadias repair. Future efforts will also involve the development of implementable care tools that will refer patients and families to appropriate experts as their physical and psychological concerns evolve over time, to support disease-specific HRQOL.

## Data Availability

The raw data supporting the conclusions of this article will be made available by the authors, without undue reservation.
